# Nomograms predict long-term survival for patients with periampullary adenocarcinoma after pancreatoduodenectomy

**DOI:** 10.1186/s12885-018-4240-x

**Published:** 2018-03-27

**Authors:** Chaobin He, Yize Mao, Jun Wang, Fangting Duan, Xiaojun Lin, Shengping Li

**Affiliations:** Department of Hepatobiliary and Pancreatic Surgery, State Key Laboratory of Oncology in South China, Collaborative Innovation Center for Cancer Medicine, Sun Yat-sen University Cancer Center, Guangzhou, Guangdong 510060 People’s Republic of China

**Keywords:** Periampullary adenocarcinoma, Pancreatoduodenectomy, Nomogram, Prediction, Prognosis

## Abstract

**Background:**

The prognosis of patients with periampullary adenocarcinoma after pancreatoduodenectomy is diverse and not yet clearly illustrated. The aim of this study was to develop a nomogram to predict individual risk of overall survival (OS) and progression-free survival (PFS) in patients with periampullary adenocarcinoma after pancreatoduodenectomy.

**Methods:**

A total of 205 patients with periampullary adenocarcinoma after pancreatoduodenectomy were retrospectively included. OS and PFS were evaluated by the Kaplan-Meier method. Two nomograms for predicting OS and PFS were established, and the predictive accuracy was measured by the concordance index (Cindex) and calibration plots.

**Results:**

Lymph node ratio (LNR), carbohydrate antigen 19–9 (CA19–9) and anatomical location were incorporated into the nomogram for OS prediction and LNR, CA19–9; anatomical location and tumor differentiation were incorporated into the nomogram for PFS prediction. All calibration plots for the probability of OS and PFS fit well. The Cindexes of the nomograms for OS and PFS prediction were 0.678 and 0.68, respectively. The OS and PFS survival times were stratified significantly using the nomogram-predicted survival probabilities.

**Conclusions:**

The present nomograms for OS and PFS prediction can provide valuable information for tailored decision-making for patients with periampullary adenocarcinoma after pancreatoduodenectomy.

## Background

The periampullary region is a complex region that is composed of distinct anatomical structures: the head of the pancreas, the distal common bile duct (CBD), the second portion of the duodenum, and the ampulla of Vater. Periampullary adenocarcinoma is now classified by the anatomic location of origin according to the 8th edition of American Joint Committee on Cancer (AJCC) staging [1]. Although periampullary adenocarcinoma accounts for approximately 0.2% of all gastrointestinal tract tumors [2] and is a relatively uncommon neoplasm, there has been an increasing trend of occurrence in recent years [3]. Periampullary adenocarcinoma is a common malignancy for which patients receive pancreaticoduodenectomy (PD), especially in Asia [4, 5]. The resectability is often limited by early local invasion of the surrounding anatomical structures, such as the superior mesenteric vein and superior mesenteric artery. The periampullary adenocarcinomas, including pancreatic head carcinoma, have a relatively low resectable rate of only 15–20% at diagnosis due to the absence of early detection methods [6, 7]. Patient survival after radical resection of adenocarcinomas of the pancreas, CBD, duodenum, and ampulla of Vater greatly varies [2, 8, 9], although some studies have reported that there is a comparatively favorable prognosis among periampullary adenocarcinomas, with 5-year overall survival (OS) rates of 30–70% after radical resection [10, 11].

Some reports have shown survival differences among different kinds of periampullary adenocarcinomas [2, 12]. Howe et al. reported that there was a higher resection rate, a lower recurrence rate, and a better OS rate for ampullary carcinomas compared with other periampullary adenocarcinomas [13]. However, pancreatic head carcinoma has been reported to have a poor prognosis even after curative therapy [14]. Anatomic location seems to provide some prognostic information in resected periampullary adenocarcinomas. Additionally, whether the ratio of lymph node (LN) with metastasis is or is not a predictor of OS in patients with periampullary adenocarcinomas has been controversial in recent years. Some studies [15, 16] suggested that the ratio of LN with metastasis was a strong predictor of OS in patients with periampullary adenocarcinomas while some reports [17, 18] failed to show that. Currently, the predictive value of variables is still uncertain. There is a lack of a staging system and consensus regarding specific risk profiles for OS and progression-free survival (PFS) in patients with periampullary adenocarcinomas; this lack makes appropriate risk stratification and physician-patient communication challenging. Risk equations and risk functions are widely applied in patient management, especially for predicting survival outcomes. Given these risk analyses and the current interest in precision medicine, it is necessary to establish prognostic tools to identify patients at risk of long-term survival and optimize patients’ selection for appropriate treatment therapy.

A nomogram, which has been developed for various cancers [19–21], is a simple graphical presentation of a multivariate predictive model showing the impact of each included variable on an outcome of interest that provides a numerical probability of the outcome [22]. Some reports have demonstrated some prognostic factors for the survival of patients with periampullary adenocarcinomas, although these prognostic factors were analyzed separately in different cohorts [12, 23]. Further, nomograms, which are capable of utilizing multiple prognostic variables, can provide a single numerical estimate of survival and an individualized prediction of survival. Unfortunately, nomograms have only been available for pancreatic carcinoma [24, 25], and few studies [12, 26] have reported nomograms for ampullary carcinoma; these studies were based on a small cohorts or without relative high concordance indexes (C-index), indicating that these nomograms were not better choices compared with the tumor-node-metastasis (TNM) stage system. Additionally, there is a lack of specific nomograms that can predict long-term survival outcomes for patients with periampullary adenocarcinomas. In the present study, we constructed nomograms from a cohort study of patients with periampullary adenocarcinoma after pancreaticoduodenectomy to predict OS and PFS.

## Methods

### Patients

Consecutive patients with newly pathologically proven periampullary adenocarcinoma after pancreatoduodenectomy carried out between February 2009 and September 2016 at the Department of Hepatobiliary and Pancreatic Surgery of Sun Yat-sen University Cancer Center were enrolled into this study. Exclusion criteria are as follows: (1) patients with major vascular invasion (superior mesenteric vein, superior mesenteric artery, or inferior vena cava) (*n* = 28); (2) patients who underwent limited surgery (e.g. ampullectomy) (*n* = 5); (3) microscopic or macroscopic incomplete resection (*n* = 2); (4) patients diagnosed with distant metastasis with or without palliative therapy (*n* = 25); (5) pathologic cell types was not adenocarcinoma (*n* = 65); (6) patients diagnosed with other concurrent primary tumors (*n* = 12); (7) lost to follow-up (*n* = 18). All patients were followed up for at least 1 year after treatment. A total of 205 patients were included for this study.

### Clinical data collection

All clinical and pathological data for diagnosis were retrieved from medical records archived at Sun Yat-sen University Cancer Center. The following clinical and pathological data were collected and analyzed: age, gender, white blood cell (WBC) count, C-reactive protein (CRP), alanine transaminase (ALT), aspartate aminotransferase (AST), albumin (ALB), total bilirubin (TBIL), alkaline phosphatase (ALP), serum levels of Carbohydrate antigen 19–9 (CA19–9), anatomical location, tumor differentiation, tumor diameter, lymph node ratio (LNR), LN metastasis and LN total number. LNR was defined as the number of LNs with metastases divided by the total number of excised LNs. The tumor stage was categorized according to the pathological TNM staging system issued by 8th edition of AJCC [1].

### Treatment procedure

Resection was performed when there was no evidence of metastasis and no arterial involvement. A classical Whipple operation was the standard resection, which was performed for all the included patients. Regional lymphadenectomy included dissection of the LNs in the hepatoduodenal ligament along the superior mesenteric vessels, and on the surface of the pancreas. After resection, a pancreaticojejunostomy, hepaticojejunostomy and gastrojejunostomy were performed. After anastomotic reconstruction, two or three silicone abdominal drains were left posterior to the pancreaticojejunostomy and hepaticojejunostomy.

### Follow-up

Patients were followed up at least every 2 months during the first year and every 3 months thereafter. CA19–9 test, liver ultrasonography, CT, and MRI were selectively performed as needed. Progression was defined as identification of suspicious imaging finding or biopsy-proven tumor in the tumor bed, regional LN area or distant area. OS was defined as the duration from the date of operation until death or the last follow-up. PFS was defined as the duration from the date of operation until the date when tumor progression was diagnosed or the last follow-up. The last follow-up was completed on August 31, 2017.

### Statistical analysis

SPSS version 22 software (SPSS Inc., Chicago, IL, USA) was used to analyze the data. The optimal cutoff value for LNR was determined using time-dependent receiver operating characteristic (ROC) analysis, which was performed using the package “survivalROC” in R version 3.2.5. The laboratory threshold was used as a cutoff value for other clinical data. Categorical variables were compared using the chi-square test and Fisher’s exact test. Continuous variables were compared using the two-tailed unpaired t-test or Mann–Whitney U-test.

Survival times were estimated using the Kaplan–Meier method and compared using the log-rank test. Analyses for survival curves were performed using MedCalc software version 11.4.2.0 (MedCalc, Ostend, Belgium). Univariate analysis was performed to assess significance of clinical and pathological characteristics. Multivariate analysis was performed using the Cox regression model for variables that were significantly associated with OS or PFS in the univariate analysis, and the corresponding 95% confidence intervals (CI) were calculated. Two-tailed *P* values less than 0.05 were considered statistically significant.

A nomogram was developed based on the independent risk factors identified in the multivariate analysis. A final model selection for the nomogram was performed by a backward step-down selection process using a threshold *P*-value of 0.05. The performance of the nomogram was measured by C-index and assessed by calibration curves. The C-index reflected the probability that a randomly selected patient with a lower probability of survival predicted via the nomogram died earlier than another randomly selected patient with a higher predicted probability. The calibration curves were used to compare the predicted probability with the observed probability in the study cohort. Bootstraps with 1000 resamples were used for the development of the nomogram and calibration curve to reduce the overfit bias. All statistical analyses were conducted using R software version 3.2.5 (R Development Core Team; http://www.r-project.org) and the “rms” package developed by Harrell (Harrell et al.).

## Results

### Patient characteristics

Of the 205 patients with periampullary adenocarcinoma who underwent pancreatoduodenectomy, ampullary adenocarcinoma was the most common diagnosis (123 patients, 60%), followed by pancreatic adenocarcinoma (67 patients, 32.7%) and duodenal adenocarcinoma (15 patients, 7.3%). Baseline characteristics of patients are shown in Table 1. The median age of all patients was 56.2 years (range 25–84 years). Most of the patients (128 patients, 62.4%) were men in the whole study cohort. Jaundice (TBIL ≥20.5 mmol/L) was reported most frequently in patients with ampullary adenocarcinoma (*P* = 0.045). These patients were more likely to have elevated values of CRP (*P* = 0.027), AST (*P* = 0.035), ALP (*P* = 0.010) and GGT (*P* <  0.001). The proportion of patients with large tumors was higher in the duodenal adenocarcinoma group than that in the ampullary adenocarcinoma group or pancreatic adenocarcinoma group (*P* = 0.002). All three groups were similar with respect to age, gender, WBC, ALT, ALB, tumor differentiation, LNR, LN metastasis and chemotherapy treatment. With the cutoff value of 0.17, LNR was associated with the optimal Youden index for OS and PFS prediction.

**Table 1:** The relationship between clinicolpathological factors and periampullary adenocarcinoma

Characteristic		*N*	Periampullary adenocarcinoma			*P*
			Panceatic head adenocarcinoma	Duodenal adenocarcinoma	Ampullary adenocarcinoma	
Total		205	67	15	123	
Age	< 60	127	38	10	79	0.551
	≥ 60	78	29	5	44	
Gender	Male	128	41	12	75	0.345
	Female	77	26	3	48	
WBC (× 10^9^/L)	< 10	172	58	13	101	0.695
	≥ 10	33	9	2	22	
CRP (mg/L)	< 8	58	27	4	27	0.027
	≥ 8	147	40	11	96	
ALT (U/L)	< 40	79	48	5	26	0.455
	≥ 40	156	49	10	97	
AST (U/L)	< 45	47	20	6	21	0.035
	≥ 45	158	47	9	102	
ALB (g/L)	< 35	52	11	3	38	0.080
	≥ 35	153	56	12	85	
TBIL (mmol/L)	< 20.5	38	18	4	16	0.045
	≥ 20.5	167	49	11	107	
ALP (U/L)	< 100	31	16	4	11	0.010
	≥ 100	174	51	11	112	
GGT (U/L)	< 50	25	15	4	6	< 0.001
	≥ 50	180	52	11	117	
Tumor differentiation	W	5	0	1	4	0.463
	W-M	8	4	0	4	
	M	108	31	7	70	
	M-P	63	23	6	34	
	P	21	9	1	11	
Tumor diameter (cm)	< 2	69	14	2	53	0.002
	≥ 2	136	53	13	70	
LNR	< 0.17	151	50	12	89	0.798
	≥ 0.17	54	17	3	34	
LN metastasis	Absent	116	36	9	71	0.836
	Present	89	31	6	52	
Chemotherapy	No	87	28	7	52	0.941
	Yes	118	39	8	71	

*WBC* white blood cell count, *CRP* C-reactive protein, *ALT* alanine transaminase, *AST* aspartate aminotransferase, *ALB* albumin, *TBIL* total bilirubin, *ALP* alkaline phosphatase, *GGT* gamma-glutamyl transpeptidase, *W* well, *M* moderate, *P* poor, *W-M* well-moderate, *M-P* moderate-poor, *LN* lymph node

### OS analysis

**Fig. 1:**
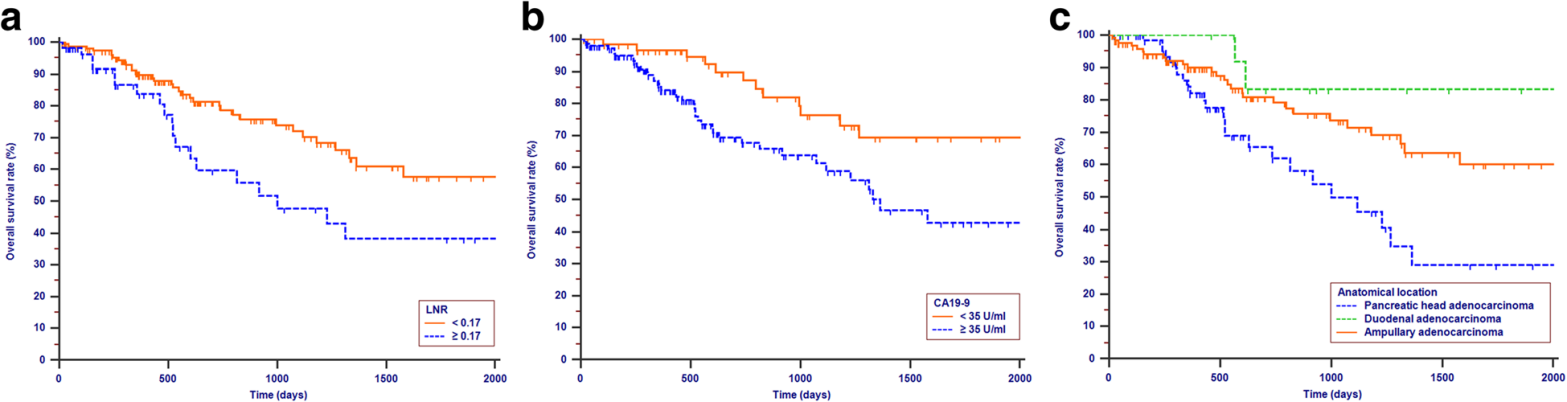
Kapaln-Meier curves for OS according to LNR (*P* = 0.015, **a** CA19–9 (*P* = 0.007, **b** and anatomical location (*P* = 0.006, **c**

The median OS time was 533 days and the 1-year, 3-year and 5-year OS rates were 88.2%, 66% and 53%, respectively. In the univariate analysis, age, gender, WBC, CRP, ALT, AST, ALB, TBIL, ALP, GGT, tumor differentiation, tumor diameter, LN metastasis, LN total number and chemotherapy treatment were not related to OS (*P* > 0.05). However, LNR, CA19–9 and anatomical location were significantly associated with OS (Table 2). These three risk factors were entered into the multivariate Cox regression analysis. After a stepwise removal of variables, LNR (HR = 1.788, 95% CI = 1.103–3.155, *P* = 0.045), CA19–9 (HR = 2.090, 95% CI = 1.082–4.037, *P* = 0.028) and anatomical location (HR = 1.892, 95% CI = 1.083–3.306, *P* = 0.025) remained significant predictors for OS (Table 3). All the included patients were further stratified by LNR (*P* = 0.015, Fig. 1a), CA19–9 (*P* = 0.007, Fig. 1b) and anatomical location (*P* = 0.006, Fig. 1c) for OS analysis. The differences of OS rates were all significant.

**Table 2:** Univariate of OS and PFS in the study cohort

Characteristic		OS		PFS	
		HR (95% CI)	*P*	HR (95% CI)	*P*
Age	< 60/ 60	1.496 (0.867–2.582)	0.148	0.643 (0.362–1.141)	0.131
Gender	male/ female	1.478 (0.857–2.547)	0.160	0.738 (0.425–1.283)	0.282I
WBC (×10^9^/L)	< 10/ ≥ 10	0.951 (0.429–2.109)	0.902	1.181 (0.598–2.330)	0.632
CRP (mg/L)	< 8/ ≥ 8	0.914 (0.508–1.645)	0.765	1.096 (0.618–1.943)	0.753
ALT (U/L)	< 40/ ≥ 40	0.869 (0.478–1.580)	0.645	1.929 (0.949–3.919)	0.069
AST (U/L)	< 45/ ≥ 45	0.670 (0.376–1.193)	0.174	1.397(0.727–2.688)	0.316
ALB (g/L)	< 35/ ≥ 35	0.987 (0.535–1.819)	0.966	0.747(0.430–1.299)	0.302
TBIL (mmol/L)	< 20.5/ ≥20.5	0.772 (0.419–1.422)	0.406	2.106 (0.957–4.634)	0.064
ALP (U/L)	< 100/ ≥ 100	0.761 (0.382–1.156)	0.438	1.73(0.744–4.023)	0.203
GGT (U/L)	< 50/ ≥ 50	0.571 (0.294–1.109)	0.098	0.906(0.430–1.909)	0.796
CA19–9 (U/ml)	< 35/ ≥ 35	2.362 (1.235–4.159)	0.009	2.095(1.147–3.829)	0.016
Tumor diffrerntiation	*W*/W-M/M/M-H/H	1.005 (0.976–1.035)	0.74	1.030(1.005–1.057)	0.020
Tumor diameter (cm)	< 2/≥ 2	0.991 (0.557–1.765)	0.976	1.486(0.828–2.666)	0.184
LNR	< 0.17/≥ 0.17	1.982 (1.13–3.479)	0.017	1.880(1.098–3.220)	0.021
LN metastasis	Absent / Present	1.408 (0.819–2.423)	0.216	2.328 (1.391–3.895)	0.001
LN total numbers	< 12/≥ 12	0.755 (0.430–1.324)	0.327	0.802 (0.478–1.344)	0.402
Anatomical location	Pancreatic/Duodenal/ Ampullary	2.287(1.325–3.948)	0.003	2.542 (1.517–4.262)	< 0.001
Chemotherapy	No/Yes	1.176(0.682–2.027)	0.559	1.713(1.006–2.915)	0.051

*HR* hazard ratio, *CI* confidence interval

Other abbreviations as in Table 1

**Table 3:** Multivariate of OS and PFS in the study cohort

Characteristic		OS		PFS	
		HR (95% CI)	*P*	HR (95% CI)	*P*
CA19–9 (U/ml)	< 35/ ≥ 35	2.090 (1.082–4.037)	0.028	1.863(1.010–3.436)	0.046
Tumor diffrerntiation	W/W-M/M/M-H/H		NI	1.031(1.005–1.058)	0.019
LNR	< 0.17/≥ 0.17	1.788 (1.103–3.155)	0.045	1.883(1.094–3.242)	0.022
Anatomical location	Pancreatic/Duodenal/ Ampullary	1.892(1.083–3.306)	0.025	1.545 (1.172–2.036)	0.002

*NI* not include

Other abbreviations as in Table 1

### PFS analysis

**Fig. 2:**
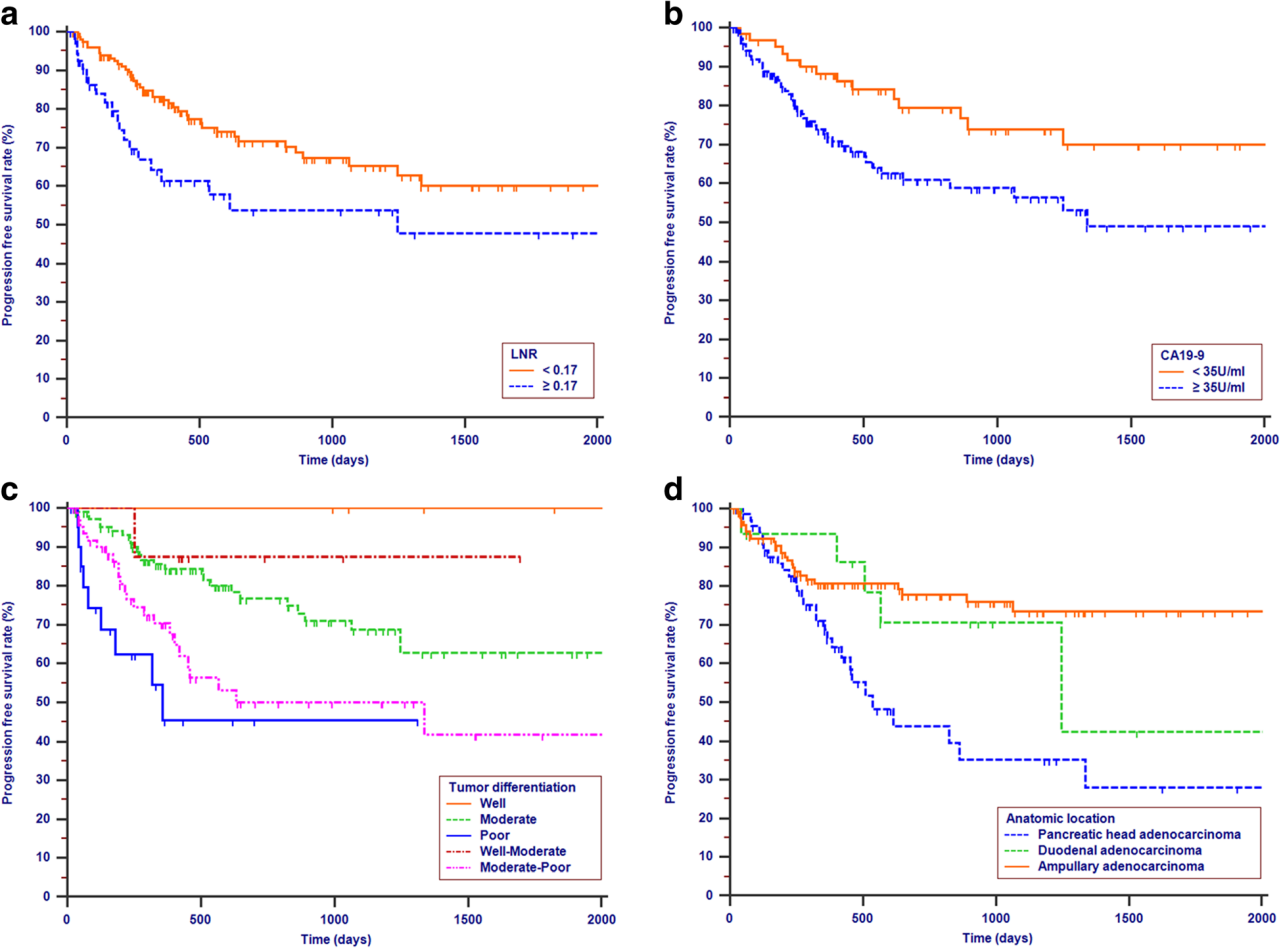
Kapaln-Meier curves for PFS according to LNR (*P* = 0.019, **a** CA19–9 (*P* = 0.014, **b** Tumor differentiation (*P* = 0.001, **c** and anatomical location (*P* = 0.001, **d**

Tumor progression was observed in 60 (29.3%) patients in the study cohort. The median progression time was 418 days. The 1-year, 3-year and 4-year OS rates were 77.2%, 62.6% and 57.1%, respectively. The univariate analysis revealed that LNR, CA19–9, tumor differentiation, LN metastasis and anatomical location were all associated with PFS (*P* <  0.05, Table 2). Multivariate analysis was then performed to delineate various prognostic indicators. Variables that were significantly associated with survival status in the univariate Cox analyses were included in the multivariate analysis. To avoid multicollinearity, the LN metastasis was not included in the multivariate analysis, as the LNR accounted for the absence or presence of LN metastasis. After adjusting for other risk factors the multivariate analysis showed that LNR (HR = 1.883, 95% CI = 1.094–3.242, *P* = 0.022), CA19–9 (HR = 1.863, 95% CI = 1.010–3.436, *P* = 0.046), tumor differentiation (HR = 1.031, 95% CI = 1.005–1.058, *P* = 0.019) and anatomical location (HR = 1.545, 95% CI = 1.172–2.036, *P* = 0.002) all remained independently associated with PFS. Additionally, LNR, CA19–9 and anatomical location were all independent predictive factors for both OS and PFS (Table 3). All the included patients were further stratified by LNR (*P* = 0.019, Fig. 2a), CA19–9 (*P* = 0.014, Fig. 2b), tumor differentiation (*P* = 0.001, Fig. 2c) and anatomical location (*P* = 0.001, Fig. 2d), respectively for PFS analysis. The differences of PFS rates were all significant.

### Construction and validation of nomograms

**Fig. 3:**
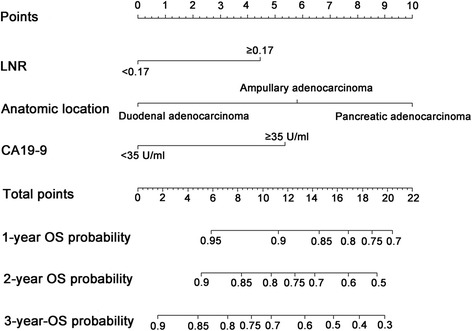
Nomogram-predicted probabilities of 1-, 2-, and 3-years OS of patients with periampullary adenocarcinoma after pancreatoduodenectomy. The nomogram is used by adding up the points identified on the scale for three or four variables. The sum is located on the “Total points” scale, and a line is drawn downward to the survival axes to determine the probability of 1-, 2-, and 3-years OS

**Fig. 4:**
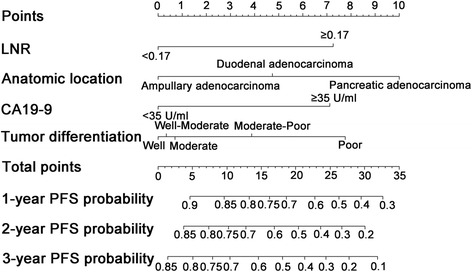
Nomogram-predicted probabilities of 1-, 2-, and 3-years PFS of patients with periampullary adenocarcinoma after pancreatoduodenectomy

**Fig. 5:**
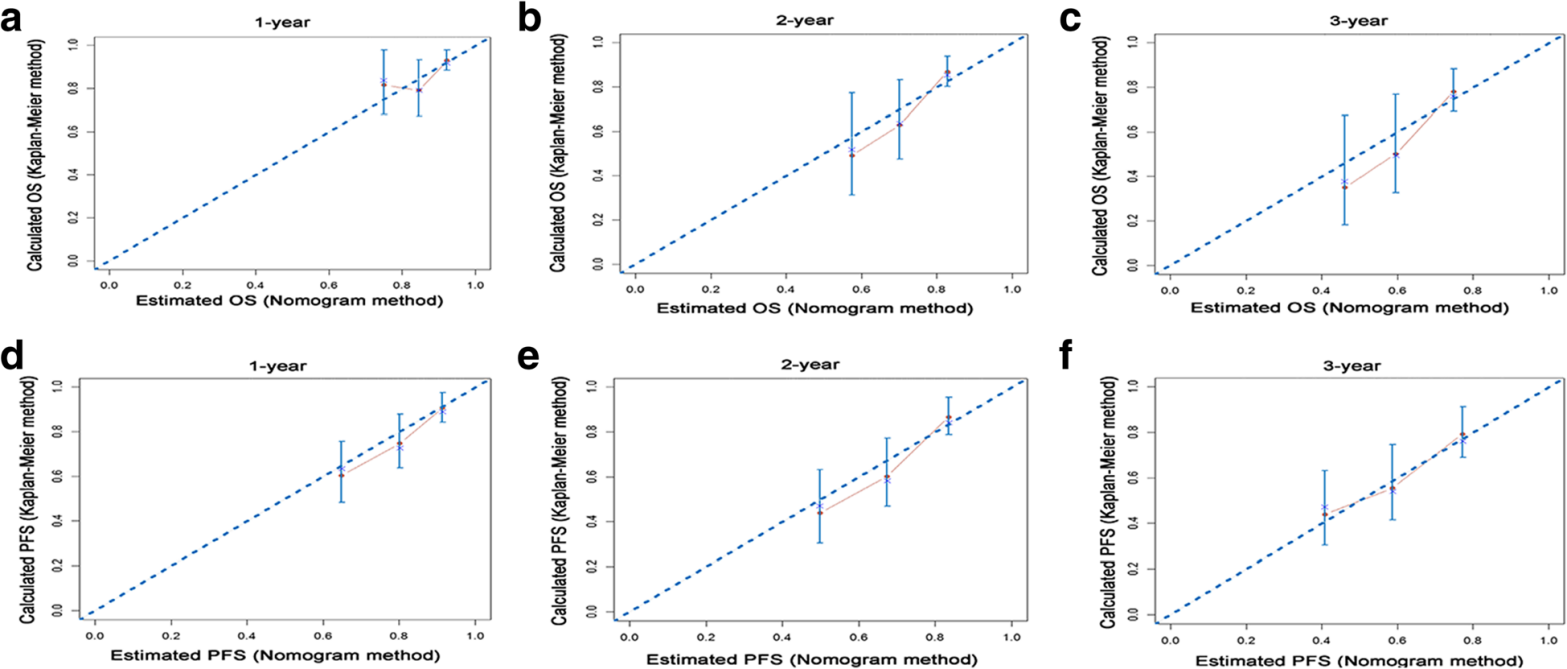
The calibration plots of the nomogram set for 1-, 2-, and 3- years OS (**a**, **b**, **c**) and PFS (**d**, **e**, **f**) prediction. X-axis represents the nomogram-predicted probability of survival; Y-axis represents the actual OS or PFS probability estimated using the Kaplan-Meier method. The diagonal line indicates the ideal nomogram reference. The line containing error bars (95% CI) represents the performance for predicting OS or PFS of the nomogram applied to the study cohort

All of the independent predictors of OS and PFS of patients in the study cohort were integrated into the nomogram (OS, Fig. 3; PFS, Fig. 4). The nomogram demonstrated good accuracy for OS prediction, with a C-index of 0.678 (95% CI = 0.612–0.744). Calibration plots for the probabilities of 1-, 2-, and 3-year OS showed fair agreement between the nomogram-predicted survival and the observed survival (Fig. 5a, b, c). The nomogram for PFS (Fig. 4) prediction was generated via the Cox proportional hazards model including the above-mentioned variables that were independently associated with PFS. The C-index for PFS prediction was 0.680 (95% CI = 0.617–0.743). Calibration plots for the probabilities of 1-, 2-, and 3-years PFS showed an optimal agreement between prediction by the nomogram and the actual observation (Fig. 5d, e, f, respectively). Additionally, the bias-corrected C-indexes of the established nomograms were higher than those of the TNM 8th stage system for both OS and PFS analyses: OS = 0.678 (95% CI = 0.612–0.744) vs OS = 0.510 (95% CI = 0.429–0.591; *P* <  0.001); PFS = 0.680 (95% CI = 0.617–0.743) vs PFS = 0.634 (95% CI = 0.566–0.702; *P* = 0.042).

### Survival analysis according to the risk stratification based on the nomogram

**Fig. 6:**
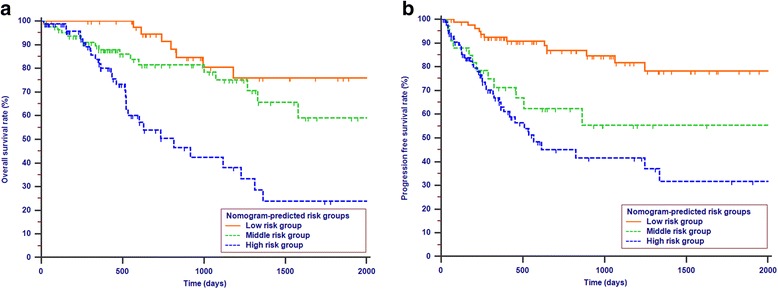
Kaplan-Meier survival curves for OS (**a**) or PFS (**b**) according to the risk levels of nomogram-predicted survival probabilities (*P* < 0.001)

All the patients in this study were categorized into several risk groups according to the probability score calculated by the nomogram. Patients with the probability score of < 10, 10–15 and ≥ 15 were assigned into the low risk group, middle risk group and high risk group, respectively. Figure 6 shows the Kaplan-Meier survival curves separated by nomogram-based grouping. The OS rates and PFS rates of patients in the low risk group were significantly higher than those of patients in the high risk group (*P* <  0.001).

## Discussion

The annual incidence of periampullary adenocarcinoma is steadily on the rise [3]. The only curative therapy for periampullary adenocarcinoma is surgical resection, usually performed as PD; the curative resectable rate is only 20% [27]. The long-term survival rate of periampullary adenocarcinoma is low and varies in a wide range among adenocarcinomas of different anatomical locations in the periampullary region [8]. Additionally, the long-term survival rate is greatly influenced by the rate of early progression [28, 29]. In this study, we identified 205 patients who underwent pancreatoduodenectomy for periampullary adenocarcinoma and grouped them by different anatomical locations. We found that ampullary adenocarcinoma constituted a relatively large proportion (60%) of all adenocarcinomas of the periampullary region, which was equivalent to the results from other studies [4, 13]. It is possible that the higher rate of resectability of ampullary adenocarcinoma at diagnosis, specified in the literature as up to 80%, which is significantly higher than that for pancreatic adenocarcinoma (20%) [2, 30], contributes to this situation. Jaundice, which is caused by the exophytic growth pattern of the tumor, was more frequently occurring in patients with ampullary adenocarcinoma in this study. This result may also partly explain the higher resectability rate of ampullary adenocarcinoma [31]. The low probability of jaundice could also lead to the late detection of patients with duodenal adenocarcinoma, along with relatively larger tumors. Further, inflammation-based markers, such as AST, ALP, GGT and CRP, are more likely to be elevated in patients with jaundice [32], similarly to our study.

The prognostic factors of patients with pancreatic adenocarcinoma [15, 33], ampullary adenocarcinoma [17], or duodenal adenocarcinoma [34] have been reported in several studies. There are few reports that focus on the prognostic factors and survival predictive systems for patients with periampullary adenocarcinoma. By using a relative large patient cohort, we found that LNR, CA19–9 and anatomical location were independently predictive factors for both OS and PFS. Lower tumor differentiation was also associated with poorer PFS. Compared with pancreatic adenocarcinoma and ampullary adenocarcinoma, patients with duodenal adenocarcinoma had higher OS rates. The PFS rates were the highest for patients with ampullary adenocarcinoma in the study cohort, which is similar to the results of other studies [13, 31]. However, chemotherapy was not an independent predictor for either OS or PFS. The need for chemotherapy after surgery was determined by a surgeon in cases with poor prognostic factors, such as LN metastasis. It is possible that the independent significance of chemotherapy was affected by the selective administration in this study. In addition, we developed a nomogram as an easy-to-apply model to predict the individual survival risk of patients with periampullary adenocarcinoma after pancreatoduodenectomy in the current study. For these patients, the nomogram internally showed that the prognosis would be better in terms of OS for patients with duodenal adenocarcinoma after pancreatoduodenectomy compared with the other two adenocarcinomas. For patients with a specific kind of periampullary adenocarcinoma, the nomogram can serve as a quantitative scoring system to estimate OS and PFS.

All variables in the nomogram were clinical or pathological characteristics. By treating continuous variables, including CA19–9 and LNR, as binary predictors, this nomogram provided a simple and visual friendly method for prognosis estimation. Further, current nomograms allow the visual tracing of the estimated risk and impact on risk when various modifiable risk factors are added or removed. Of all variables included in the nomagram, CA199–9 was previously accepted as a prognostic factor for OS and PFS of patients with ampullary adenocarcinoma [23, 35, 36]. With the cutoff value of 35 U/ml, patients can be easily separated into groups with elevated values or normal values of CA19–9. Previous reports have shown that patients with higher preoperative CA19–9 levels were more likely to have higher tumor burdens and reduced chances of survival [37]. In the current study, we found that patients with elevated CA19–9 levels were more likely to have reduced OS and PFS. Our results are similar to the study by Cristina et al. [23], which showed that a lower preoperative CA19–9 level correlated not only with a lower pathologic stage but also with an increased postoperative survival.

LNR was demonstrated as a predictor of survival by many reports [12, 26]. The present study showed that a higher LNR was significantly associated with poorer OS and PFS. Our study did not show an association between the total number of resected nodes and OS or PFS in patients with periampullary adenocarcinoma. This result was similar to other reports [38, 39] in which the total number of nodes examined was not a predictive factor for survival. Furthermore, as demonstrated in the present study [16, 40], it was found that LNR was a superior predictor of survival compared with the total number of resected LNs. AJCC recommends at least 12 harvested nodes for accurate staging because insufficient LNs may lead to under-stage the N category in many kinds of tumors, such as gastric carcinoma or pancreatic carcinoma [1, 41, 42]. A patient with fewer resected LNs may have a decreased survival. The elevated LNR was a sign that showed the tendency of metastasis or progression and was associated with the poorer OS and PFS in this study. Our study demonstrated results similar to previous research [43] conducted by the MD Anderson Cancer Center, in which the strong association between high LNR (> 0.15) and low distant metastasis-free survival was detected.

OS and PFS of patients with adenocarcinoma from different anatomical locations in the periampullary region were analyzed and compared. Some studies [2, 44, 45] reported the 5-year OS rates of duodenal adenocarcinoma, ampullary adenocarcinoma and pancreatic carcinoma were 59%, 39% and 15%, respectively. As in the previous studies, our study showed that patients with duodenal adenocarcinoma had a better long-term OS. Interestingly, patients with ampullary adenocarcinoma kept an even higher long-term PFS rate compared with duodenal adenocarcinoma and pancreatic adenocarcinoma. Bucher et al. [31] also reported that ampullary carcinomas had a lower recurrence rate, which was in contrast with other carcinomas of the periampullary region. It is possible that the adenoma-to-carcinoma sequence of ampullary carcinoma, which is similar to colon carcinoma, contributed to this phenomenon [46]. In addition to the anatomical location, other pathological characteristics, such as tumor differentiation, have been reported to be associated with the progression of periampullary carcinoma [47, 48]. Park et al. [49] revealed that tumor differentiation was a significant influencing factor of early progression in patients with periampullary carcinoma. In our multivariate analysis, we also found that poor tumor differentiation was a poor prognostic factor for PFS. In general, it should seem that OS rate would be reduced in patients with poor tumor differentiation [50, 51], and our data showed such a tendency, but failed to show statistical significance, possibly due to the small total number of patients in the study. Tumor differentiation as a prognostic factor for PFS needs to be further elucidated by future prospective randomized controlled studies.

All the variables included in the nomograms were significant predictive factors for OS and FPS in this study. Our nomograms showed good C-indexes in the study cohort. The C-indexes of the nomograms for OS and PFS prediction were 0.678 and 0.680, respectively. This means that if two patients with different nomogram points are selected, the probabilities that the patient with the higher nomogram score would die earlier and of which the disease would progress earlier are both over 67%. Further, the comparison of C-indexes showed that the established nomogram displayed more powerful efficiency of discrimination for both OS and CSS prediction compared with the TNM 8th edition stage system. Calibration plots show how accurate the results are as predicted by the nomogram model compared with the results estimated by the Kaplan-Meier method. The calibration plots of internal validation demonstrated good fitness for OS and PFS prediction, as the predicted survival probabilities at 1-, 2-, and 3-years estimated via the nomogram were closely aligned with the actual survival times. Additionally, a clear risk stratification of survival times using nomogram-predicted survival probabilities was demonstrated by survival curves. Therefore, a user-friendly nomogram can help physicians to predict the prognosis of patients and provide individualized treatment.

There were several limitations in this study. First, it was a retrospective study that relied on a single-institutional dataset. Geographic and institutional heterogeneity of patients may affect these results. Second, some of the potential predictive variables could not be included into the nomograms. Third, an external validation for predictive accuracy of the nomograms was not conducted in our study, which decreased the applicability of the nomograms to an external cohort. Large prospective studies are needed to further validate the accuracy of these prognostic nomograms.

## Conclusions

The predictive power of variables for survival prediction in patients was analyzed in this study. This was the first study that included LNR and anatomical location to nomograms for predicting OS and PFS in patients with periampullary adenocarcinoma after pancreatoduodenectomy, potentially facilitating highly tailored patient management.

## Abbreviations

ALB: Albumin
ALP: Alkaline phosphatase
ALT: Alanine transaminase
AST: Aspartate aminotransferase
CA19–9: Carbohydrate antigen 19–9
CBD: Distal common bile duct
CI: Confidence interval
C-index: Concordance index
CRP: C-reactive protein
GGT: Gamma-glutamyl transpeptidase
LN: Lymph node
LNR: Lymph node ratio
OS: Overall survival
PD: Pancreaticoduodenectomy
PFS: Progression free survival
ROC: Receiver operating characteristic
TBIL: Total bilirubin
TNM: Tumor-node-metastasis
WBC: White blood cell

## References

[1] Amin MBES, Greene F (2017). AJCC Cancer staging manual.

[2] Yeo CJ, Sohn TA, Cameron JL, Hruban RH, Lillemoe KD, Pitt HA (1998). Periampullary adenocarcinoma: analysis of 5-year survivors. Ann Surg.

[3] Siegel RL, Miller KD, Jemal A (2016). Cancer statistics, 2016. CA Cancer J Clin.

[4] Nakase A, Matsumoto Y, Uchida K, Honjo I (1977). Surgical treatment of cancer of the pancreas and the periampullary region: cumulative results in 57 institutions in Japan. Ann Surg.

[5] Petrova E, Ruckert F, Zach S, Shen Y, Weitz J, Grutzmann R, Wittel UA, Makowiec F, Hopt UT, Bronsert P (2017). Survival outcome and prognostic factors after pancreatoduodenectomy for distal bile duct carcinoma: a retrospective multicenter study. Langenbeck's Arch Surg.

[6] Westgaard A, Tafjord S, Farstad IN, Cvancarova M, Eide TJ, Mathisen O, Clausen OP, Gladhaug IP (2008). Pancreatobiliary versus intestinal histologic type of differentiation is an independent prognostic factor in resected periampullary adenocarcinoma. BMC Cancer.

[7] Herreros-Villanueva M, Hijona E, Cosme A, Bujanda L (2012). Adjuvant and neoadjuvant treatment in pancreatic cancer. World J Gastroenterol.

[8] van Geenen RC, van Gulik TM, Offerhaus GJ, de Wit LT, Busch OR, Obertop H, Gouma DJ (2001). Survival after pancreaticoduodenectomy for periampullary adenocarcinoma: an update. Eur J Surg Oncol.

[9] Jang JY, Kim SW, Park DJ, Ahn YJ, Yoon YS, Choi MG, Suh KS, Lee KU, Park YH (2005). Actual long-term outcome of extrahepatic bile duct cancer after surgical resection. Ann Surg.

[10] Narang AK, Miller RC, Hsu CC, Bhatia S, Pawlik TM, Laheru D, Hruban RH, Zhou J, Winter JM, Haddock MG (2011). Evaluation of adjuvant chemoradiation therapy for ampullary adenocarcinoma: the Johns Hopkins Hospital-Mayo Clinic collaborative study. Radiat Oncol.

[11] Kim K, Chie EK, Jang JY, Kim SW, Oh DY, Im SA, Kim TY, Bang YJ, Ha SW (2009). Role of adjuvant chemoradiotherapy for ampulla of Vater cancer. Int J Radiat Oncol Biol Phys.

[12] Tol JA, Brosens LA, van Dieren S, van Gulik TM, Busch OR, Besselink MG, Gouma DJ (2015). Impact of lymph node ratio on survival in patients with pancreatic and periampullary cancer. Br J Surg.

[13] Howe JR, Klimstra DS, Moccia RD, Conlon KC, Brennan MF (1998). Factors predictive of survival in ampullary carcinoma. Ann Surg.

[14] Seppanen H, Juuti A, Mustonen H, Haapamaki C, Nordling S, Carpelan-Holmstrom M, Siren J, Luettges J, Haglund C, Kiviluoto T (2017). The results of pancreatic resections and long-term survival for pancreatic ductal adenocarcinoma: a single-institution experience. Scand J Surg.

[15] Gonen M, Jarnagin WR, D'Angelica M, DeMatteo RP, Fong Y, Brennan MF, Allen PJ, House MG (2007). Prognostic significance of pathologic nodal status in patients with resected pancreatic cancer. J Gastrointest Surg.

[16] Pawlik TM, Gleisner AL, Cameron JL, Winter JM, Assumpcao L, Lillemoe KD, Wolfgang C, Hruban RH, Schulick RD, Yeo CJ (2007). Prognostic relevance of lymph node ratio following pancreaticoduodenectomy for pancreatic cancer. Surgery.

[17] Pomianowska E, Westgaard A, Mathisen O, Clausen OP, Gladhaug IP (2013). Prognostic relevance of number and ratio of metastatic lymph nodes in resected pancreatic, ampullary, and distal bile duct carcinomas. Ann Surg Oncol.

[18] Hurtuk MG, Hughes C, Shoup M, Aranha GV (2009). Does lymph node ratio impact survival in resected periampullary malignancies?. Am J Surg.

[19] He CB, Lao XM, Lin XJ (2017). Transarterial chemoembolization combined with recombinant human adenovirus type 5 H101 prolongs overall survival of patients with intermediate to advanced hepatocellular carcinoma: a prognostic nomogram study. Chin J Cancer.

[20] Kattan MW, Karpeh MS, Mazumdar M, Brennan MF (2003). Postoperative nomogram for disease-specific survival after an R0 resection for gastric carcinoma. J Clin Oncol.

[21] Kawai K, Ishihara S, Yamaguchi H, Sunami E, Kitayama J, Miyata H, Watanabe T (2015). Nomogram prediction of metachronous colorectal neoplasms in patients with colorectal cancer. Ann Surg.

[22] Fu YP, Ni XC, Yi Y, Cai XY, He HW, Wang JX, Lu ZF, Han X, Cao Y, Zhou J (2016). A novel and validated inflammation-based score (IBS) predicts survival in patients with hepatocellular carcinoma following curative surgical resection: a STROBE-compliant article. Medicine.

[23] Ferrone CR, Finkelstein DM, Thayer SP, Muzikansky A, Fernandez-delCastillo C, Warshaw AL (2006). Perioperative CA19-9 levels can predict stage and survival in patients with resectable pancreatic adenocarcinoma. J Clin Oncol.

[24] Deng QL, Dong S, Wang L, Zhang CY, Ying HF, Li ZS, Shen XH, Guo YB, Meng ZQ, Yu JM (2017). Development and validation of a nomogram for predicting survival in patients with advanced pancreatic ductal adenocarcinoma. Sci Rep.

[25] Song W, Miao DL, Chen L (2018). Nomogram for predicting survival in patients with pancreatic cancer. OncoTargets and therapy.

[26] Kwon J, Kim K, Chie EK, Kim BH, Jang JY, Kim SW, Oh DY, Bang YJ (2017). Prognostic relevance of lymph node status for patients with ampullary adenocarcinoma after radical resection followed by adjuvant treatment. Eur J Surg Oncol.

[27] Kamisawa T, Wood LD, Itoi T, Takaori K (2016). Pancreatic cancer. Lancet (London, England).

[28] Fischer R, Breidert M, Keck T, Makowiec F, Lohrmann C, Harder J (2012). Early recurrence of pancreatic cancer after resection and during adjuvant chemotherapy. Saudi J Gastroenterol.

[29] Shimada K, Sakamoto Y, Sano T, Kosuge T (2006). The role of paraaortic lymph node involvement on early recurrence and survival after macroscopic curative resection with extended lymphadenectomy for pancreatic carcinoma. J Am Coll Surg.

[30] Kim RD, Kundhal PS, McGilvray ID, Cattral MS, Taylor B, Langer B, Grant DR, Zogopoulos G, Shah SA, Greig PD (2006). Predictors of failure after pancreaticoduodenectomy for ampullary carcinoma. J Am Coll Surg.

[31] Bucher P, Chassot G, Durmishi Y, Ris F, Morel P (2007). Long-term results of surgical treatment of Vater's ampulla neoplasms. Hepato-Gastroenterology.

[32] Jin H, Pang Q, Liu H, Li Z, Wang Y, Lu Y, Zhou L, Pan H, Huang W (2017). Prognostic value of inflammation-based markers in patients with recurrent malignant obstructive jaundice treated by reimplantation of biliary metal stents: a retrospective observational study. Medicine.

[33] Bhatti I, Peacock O, Awan AK, Semeraro D, Larvin M, Hall RI (2010). Lymph node ratio versus number of affected lymph nodes as predictors of survival for resected pancreatic adenocarcinoma. World J Surg.

[34] Ecker BL, McMillan MT, Datta J, Lee MK, Karakousis GC, Vollmer CM, Drebin JA, Fraker DL, Roses RE (2017). Adjuvant chemotherapy versus chemoradiotherapy in the management of patients with surgically resected duodenal adenocarcinoma: a propensity score-matched analysis of a nationwide clinical oncology database. Cancer.

[35] Okano K, Oshima M, Yachida S, Kushida Y, Kato K, Kamada H, Wato M, Nishihira T, Fukuda Y, Maeba T (2014). Factors predicting survival and pathological subtype in patients with ampullary adenocarcinoma. J Surg Oncol.

[36] Kurihara C, Yoshimi F, Sasaki K, Iijima T, Kawasaki H, Nagai H (2013). Clinical value of serum CA19-9 as a prognostic factor for the ampulla of Vater carcinoma. Hepato-Gastroenterology.

[37] Nakao A, Oshima K, Nomoto S, Takeda S, Kaneko T, Ichihara T, Kurokawa T, Nonami T, Takagi H (1998). Clinical usefulness of CA-19-9 in pancreatic carcinoma. Semin Surg Oncol.

[38] Sierzega M, Popiela T, Kulig J, Nowak K (2006). The ratio of metastatic/resected lymph nodes is an independent prognostic factor in patients with node-positive pancreatic head cancer. Pancreas.

[39] Hartwig W, Hackert T, Hinz U, Gluth A, Bergmann F, Strobel O, Buchler MW, Werner J (2011). Pancreatic cancer surgery in the new millennium: better prediction of outcome. Ann Surg.

[40] John BJ, Naik P, Ironside A, Davidson BR, Fusai G, Gillmore R, Watkins J, Rahman SH (2013). Redefining the R1 resection for pancreatic ductal adenocarcinoma: tumour lymph nodal burden and lymph node ratio are the only prognostic factors associated with survival. HPB.

[41] Datta J, Lewis RS, Mamtani R, Stripp D, Kelz RR, Drebin JA, Fraker DL, Karakousis GC, Roses RE (2014). Implications of inadequate lymph node staging in resectable gastric cancer: a contemporary analysis using the National Cancer Data Base. Cancer.

[42] Slidell MB, Chang DC, Cameron JL, Wolfgang C, Herman JM, Schulick RD, Choti MA, Pawlik TM (2008). Impact of total lymph node count and lymph node ratio on staging and survival after pancreatectomy for pancreatic adenocarcinoma: a large, population-based analysis. Ann Surg Oncol.

[43] Roland CL, Katz MH, Gonzalez GM, Pisters PW, Vauthey JN, Wolff RA, Crane CH, Lee JE, Fleming JB (2012). A high positive lymph node ratio is associated with distant recurrence after surgical resection of ampullary carcinoma. J Gastrointest Surg.

[44] Warren KW, Choe DS, Plaza J, Relihan M (1975). Results of radical resection for periampullary cancer. Ann Surg.

[45] Michelassi F, Erroi F, Dawson PJ, Pietrabissa A, Noda S, Handcock M, Block GE (1989). Experience with 647 consecutive tumors of the duodenum, ampulla, head of the pancreas, and distal common bile duct. Ann Surg.

[46] Klein F, Jacob D, Bahra M, Pelzer U, Puhl G, Krannich A, Andreou A, Gul S, Guckelberger O (2014). Prognostic factors for long-term survival in patients with ampullary carcinoma: the results of a 15-year observation period after pancreaticoduodenectomy. HPB Surg.

[47] Moriya T, Kimura W, Hirai I, Mizutani M, Ma J, Kamiga M, Fuse A (2006). Nodal involvement as an indicator of postoperative liver metastasis in carcinoma of the papilla of Vater. J Hepato-Biliary-Pancreat Surg.

[48] Talamini MA, Moesinger RC, Pitt HA, Sohn TA, Hruban RH, Lillemoe KD, Yeo CJ, Cameron JL (1997). Adenocarcinoma of the ampulla of Vater. A 28-year experience. Ann Surg.

[49] Park JS, Yoon DS, Kim KS, Choi JS, Lee WJ, Chi HS, Kim BR (2007). Factors influencing recurrence after curative resection for ampulla of Vater carcinoma. J Surg Oncol.

[50] Hornick JR, Johnston FM, Simon PO, Younkin M, Chamberlin M, Mitchem JB, Azar RR, Linehan DC, Strasberg SM, Edmundowicz SA (2011). A single-institution review of 157 patients presenting with benign and malignant tumors of the ampulla of Vater: management and outcomes. Surgery.

[51] Winter JM, Cameron JL, Olino K, Herman JM, de Jong MC, Hruban RH, Wolfgang CL, Eckhauser F, Edil BH, Choti MA (2010). Clinicopathologic analysis of ampullary neoplasms in 450 patients: implications for surgical strategy and long-term prognosis. J Gastrointest Surg.

